# The 6-min walk test in transthyretin cardiac amyloidosis: prognostic utility put to the real-world test

**DOI:** 10.3389/fmed.2025.1639586

**Published:** 2025-09-08

**Authors:** Michael Poledniczek, Anna Grubmüller, Christina Kronberger, Robin Willixhofer, Nikita Ermolaev, René Rettl, Christina Binder, Luciana Camuz Ligios, Mahshid Eslami, Bernhard Gregshammer, Luca List, Christian Nitsche, Andreas Anselm Kammerlander, Christian Hengstenberg, Johannes Kastner, Jutta Bergler-Klein, Roza Badr Eslam, Franz Duca

**Affiliations:** Division of Cardiology, Department of Internal Medicine II, Medical University of Vienna, Vienna, Austria

**Keywords:** 6-min walk test, transthyretin amyloid cardiomyopathy, exercise testing, physical performance, risk stratification

## Abstract

**Background:**

The 6-min walk test distance (6MWD) was identified as a predictor of mortality in transthyretin amyloid cardiomyopathy (ATTR-CM). However, its real-world applicability remains uncertain, as only therapy-naïve patients were included in the primary analysis.

**Methods:**

Patients from a prospective ATTR-CM registry were analyzed and included if a 6MWT was completed at baseline.

**Results:**

A total of 252 patients [79.8 years, interquartile range (IQR): 75.4–83.7] were included. After a median of 21.7 (IQR: 12.7–34.1) months, 61 (24.2%) patients died. A 6MWD of <350 m was associated with worse survival [hazard ratio (HR): 3.29, 95% confidence interval (CI): 1.94–5.55, *p* < 0.001], even after adjusting for National Amyloidosis Centre stage (HR: 2.30, 95% CI: 1.29–4.10, *p* = 0.005). The Δ6MWD thresholds of <−35 meters/−5% were only associated with mortality after adjusting for change in treatment status.

**Conclusion:**

The 6MWD is independently associated with mortality in ATTR-CM irrespective of treatment status. A baseline 6MWD of <350 m is associated with a ~3-fold risk for all-cause mortality. However, our results suggest that the Δ6MWD should only be used in patients on stable background therapy for the estimation of prognosis.

## Graphical abstract

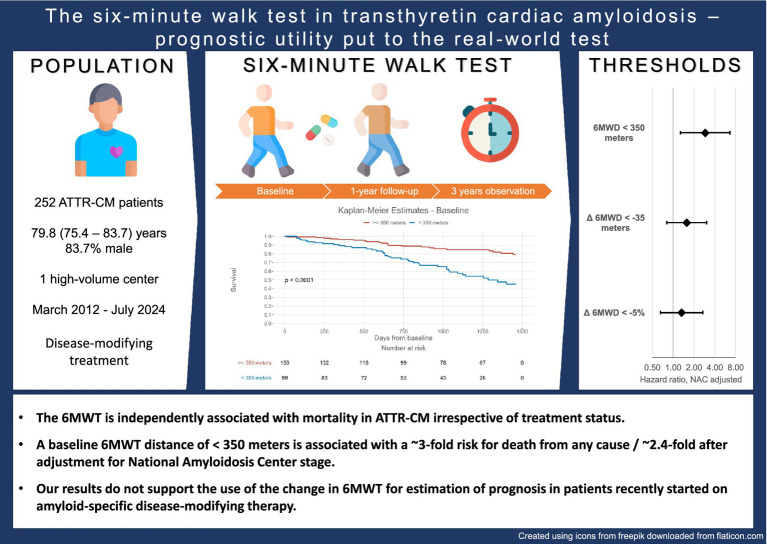

## 1 Introduction

Transthyretin amyloid cardiomyopathy (ATTR-CM) is a myocardial disease characterized by interstitial deposition of amyloid fibrils (1). As a result, myocardial wall thickness increases, and myocardial compliance is impaired (1). Due to the subsequent decrease in cardiac output, patients suffer from shortness of breath on exertion, reduced exercise capacity, and ultimately reduced quality of life (1–4). While staging of the ATTR-CM currently relies predominantly on biomarkers of heart failure (5–7), alternative assessments, e.g., exercise testing and health-related quality of life, have recently been explored as markers of adverse outcome in ATTR-CM (3, 4, 8).

The 6-min walk test (6MWT) is a relatively easy-to-use tool for assessing submaximal physical performance (9) and has demonstrated an independent association with outcomes in patients with light chain amyloid cardiomyopathy (10). Recently, these findings were replicated among patients with ATTR-CM in a large retrospective analysis by Ioannou et al. (8) However, important questions regarding the real-life applicability of these results remain unanswered. Most notably, while the disease-specific therapeutic tafamidis is widely adopted in clinical practice and has been demonstrated to ameliorate the decline in performance in the 6MWT after 6 months of treatment (11), there is currently no evidence on the prognostic utility of the 6MWT in patients treated with tafamidis or other disease-specific therapeutics, such as inotersen and patisiran.

We, therefore, aimed to evaluate the association between 6MWT distance (6MWD) and all-cause mortality in ATTR-CM patients both with and without disease-modifying drugs at baseline, assess changes at 1-year follow-up, and validate previous findings (8) in a prospective cohort of patients diagnosed with ATTR-CM.

## 2 Methods

### 2.1 Setting

The present study was performed within the scope of a prospective cardiac amyloidosis registry at the Medical University of Vienna, Department of Internal Medicine II, Division of Cardiology. The registry was approved by the local ethics committee (#1079/2023) and implemented in line with the principles outlined in the Declaration of Helsinki.

### 2.2 Subjects and study design

Consecutive patients presenting to the institution’s tertiary referral center for cardiac amyloidosis between March 2012 and July 2024 were screened for eligibility and included in the analysis if the following inclusion criteria were met: (1) a diagnosis of ATTR-CM in accordance with the cardiomyopathy guidelines by the European Society of Cardiology (12), or prior to the publication of the non-invasive diagnostic algorithm by Gillmore et al. (13), utilizing endomyocardial biopsy, (2) completion of 6MWT at baseline, and (3) provision of written informed consent. Patients were excluded from the analysis in the presence of cardiac amyloidosis other than ATTR-CM. Following diagnosis, patients were offered genetic testing to explore whether genetic variants in the transthyretin gene were causative of the disease. Genetic testing was accepted by all included patients.

### 2.3 Study parameters

The 6MWT was performed in accordance with the guidelines issued by the American Thoracic Society (14). In short, patients were asked to walk as far as they could—at their own pace—up and down a dedicated 50-m-long corridor with flat, non-slippery flooring, marked in 1-m increments and isolated from other patients, which remained unchanged throughout the study period. Time-keeping was performed by instructed clinic assistants or junior physicians (M.P., A.G., C.K., R.W., N.E., B.G., and L.L.). In addition to the walking distance, the expected distance was calculated using the formula proposed by Enright and Sherrill (15).

In addition, patient demographics, medical history, and laboratory and echocardiographic assessments were compiled at baseline. At 1-year follow-up, laboratory analysis and a repeated 6MWT were performed. All laboratory analyses were conducted at the Department of Laboratory Medicine at the Medical University of Vienna within 3 h of blood sampling. The estimated glomerular filtration rate (eGFR) was calculated using the Chronic Kidney Disease Epidemiology Collaboration formula (9).

### 2.4 Study endpoints

The primary endpoint of the present study was all-cause mortality, and patients were followed for up to 4 years. Endpoint data were assessed using (1) periodic queries on the national statistics authority’s (Statistics Austria) death registry and electronic medical records, (2) routine outpatient visits, and (3) telephone interviews with patients or patients’ relatives.

### 2.5 Statistical analysis

All categorical variables are presented as numbers and percentages, while metric parameters are presented as mean and standard deviation (SD) or median and interquartile range (IQR), depending on the respective variables’ distribution, analyzed utilizing the Shapiro–Wilk test.

Differences between the groups were assessed using the chi-square test for categorical parameters and the Kruskal–Wallis test for metric variables. For the comparison of paired parameters, the Wilcoxon signed–rank test and the McNemar test were utilized, respectively.

The patients’ baseline and follow-up characteristics were compared. Furthermore, patients were compared according to the difference (Δ) in 6MWD from baseline to follow-up. Determinants of the 6MWD were assessed using multiple linear regression analysis. The association of the 6MWD with the primary study endpoint was explored using univariable and multivariable Cox proportional hazard regression analyses. In the multivariable model, the 6MWD was adjusted for the United Kingdom National Amyloidosis Centre (NAC) stage (6). In addition to crude values, previously published cutoffs for both the 6MWD and Δ6MWD by Ioannou et al. (8) were applied. All subjects with available data points were included in the respective analyses. For analyses involving Δ values, subjects who did not complete the 1-year follow-up period were excluded from the analyses.

Statistical significance was defined as a *p*-value of ≤0.05. All statistical analyses were performed using BlueSky Statistics 10.3.4, R package version 8.95 (BlueSky Statistics LLC, Chicago, IL, USA).

## 3 Results

### 3.1 Baseline characteristics

**Figure 1:**
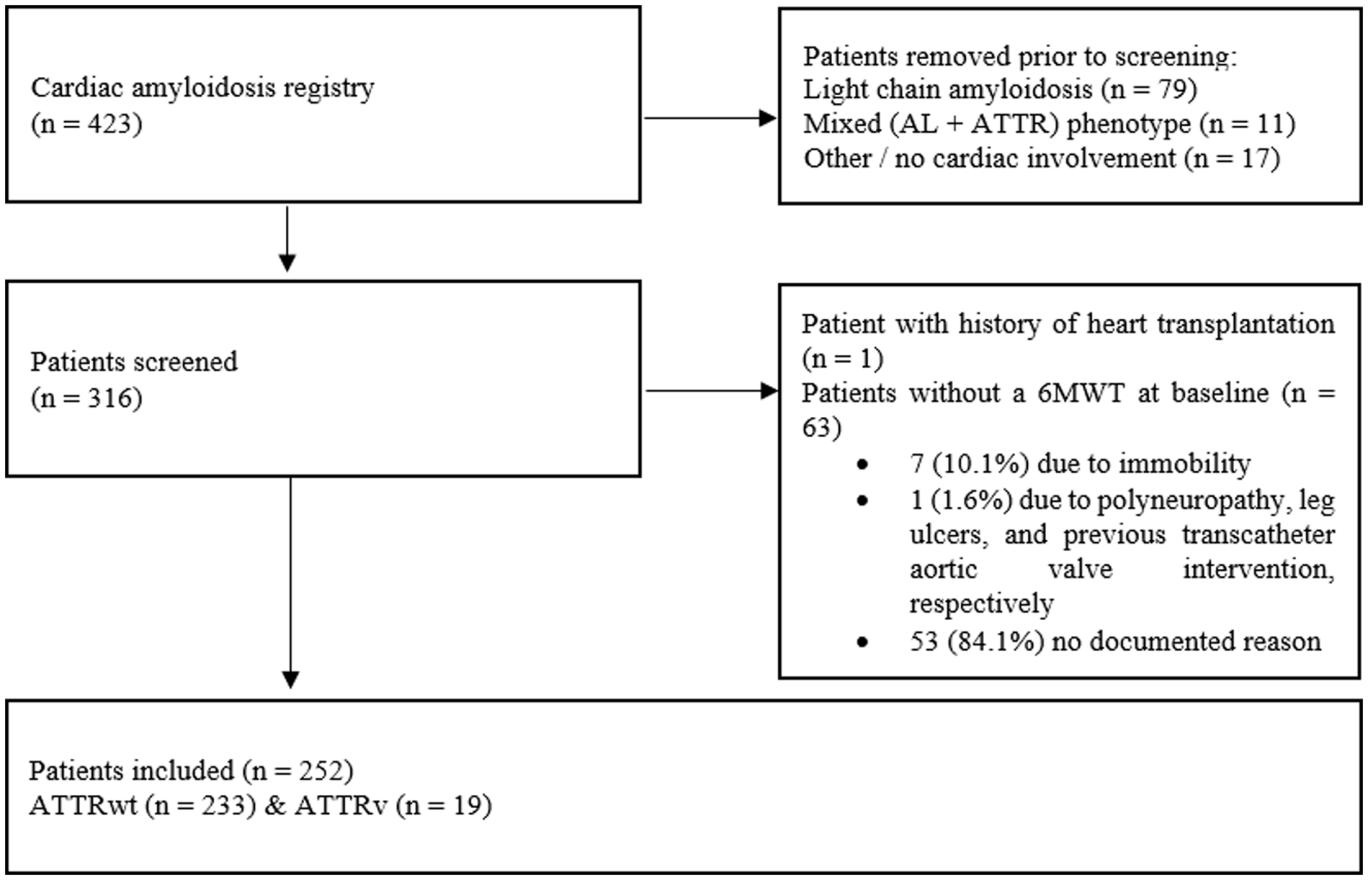
The patient recruitment process; 6MWT indicates six-minute walk test; AL, light chain amyloid; ATTR, transthyretin amyloid; ATTRv, variant transthyretin amyloid; ATTRwt, wild-type transthyretin amyloid.

A total of 316 patients were screened, and 252 patients [79.8 (IQR: 75.4–83.7) years, 83.7% male] with ATTR-CM were included in the final analysis. A total of 64 patients were excluded from the analysis. In the entire cohort, the primary endpoint of death from any cause was met by 61 patients after a median of 21.7 (IQR: 12.7–34.1) months. The detailed recruitment process is shown in Figure 1, and baseline characteristics are presented in Table 1.

**Table 1:** Patient characteristics at baseline and follow-up.

Variable	Baseline (*n* = 252)	Follow-up (*n* = 190)	*p*-value
Patient demographics			
Age, years, median (IQR)	79.8 (75.4–83.7)	80.7 (76.3–84.5)	
Male sex, *n* (%)	211 (83.7%)		
ATTRv, *n* (%)	19 (7.6%)		
6MWD, m, mean ± SD	362 ± 127	368 ± 137	0.057
6MWD threshold, *n* (%)	99 (39.3%)	70 (41.7%)	0.086
6MWD expected, m, mean ± SD	462 ± 79	458 ± 86	**<0.001**
6MWD, % of expected, mean ± SD	76.7 ± 28.9	76.7 ± 33.9	0.172
Primary endpoint, *n* (%)	61 (24.2%)	47 (24.7%)	
Time to primary endpoint, months, median (IQR)	21.7 (12.7–34.1)	11.2 (6.0–15.9)	
Clinical parameters			
BMI, kg/m^2^, median (IQR)	25.1 (23.2–27.9)	25.0 (23.0–27.4)	**<0.001**
NT-proBNP, pg./mL, median (IQR)	2,405 (1,153–4,235)	2,452 (1,307–4,910)	0.063
TnT, ng/L, median (IQR)	46.0 (29.5–69.0)	53.0 (35.0–73.0)	**0.031**
Creatinine, mg/dL, median (IQR)	1.18 (0.96–1.50)	1.28 (1.03–1.54)	**<0.001**
eGFR, mL/min/1.73 m^2^, median (IQR)	57.2 (42.3–73.0)	51.7 (38.5–65.4)	**<0.001**
Medical history			
Atrial fibrillation, *n* (%)^a^	153 (61.0%)		
Significant coronary artery disease, *n* (%)	53 (21.0%)		
Arterial hypertension, *n* (%)^b^	176 (70.4%)		
Diabetes mellitus, *n* (%)^b^	51 (20.4%)		
Intervention for valvular heart disease, *n* (%)			
Aortic valve	23 (9.1%)		
Mitral valve	8 (3.2%)		
Tricuspid valve	3 (1.2%)		
Device, *n* (%)	48 (19.0%)		
ICD	0 (0.0%)		
PM	38 (15.1%)		
CRT	10 (4.0%)		
NYHA, *n* (%)^b^^,^^f^			**0.001**
1	40 (16.1%)	40 (22.6%)	
2	122 (49.2%)	92 (52.0%)	
3	86 (34.7%)	45 (25.4%)	
NAC, *n* (%)^b^^,^^e^			0.874
1	128 (51.2%)	85 (46.4%)	
2	74 (29.6%)	59 (32.2%)	
3	48 (19.2%)	39 (21.3%)	
Medical therapy			
Tafamidis, *n* (%)^b^	13 (5.2%)	159 (84.6%)	**<0.001**
Patisiran, *n* (%)^b^	0 (0.0%)	2 (1.1%)	/
Inotersen, *n* (%)^b^	0 (0.0%)	2 (1.1%)	/
Time to disease-modifying therapy, days, median (IQR)	72 (42–126)		
Loop diuretics, *n* (%)^a^^,^^d^	132 (52.6%)	123 (66.5%)	**0.005**
Loop diuretics dose, mg/kg, median (IQR)^a^^,^^c^	0.000 (0.000–0.502)	0.253 (0.000–0.667)	**0.002**
MRA, *n* (%)^a^^,^^c^	117 (46.6%)	93 (50.0%)	0.396
SGLT-2i, *n* (%)^a^^,^^c^	45 (17.9%)	34 (18.3%)	**<0.001**
Beta receptor antagonists, *n* (%)^a^^,^^c^	127 (50.6%)	81 (43.5%)	**0.006**

6MWD indicates 6-min walk test distance; ATTRv, variant transthyretin amyloid; BMI, body mass index; BP, blood pressure; bpm, beats per minute; CRT, cardiac resynchronisation pacemaker; eGFR, estimated glomerular filtration rate; ICD, implanted cardioverter-defibrillator; IQR, interquartile range; mmHg, millimeters of mercury; MRA, mineralocorticoid receptor antagonist; NAC, United Kingdom National Amyloidosis Centre staging system; NT-proBNP, N-terminal prohormone of B-type natriuretic peptide; NYHA, New York Heart Association stage; PM, pacemaker; SD, standard deviation; SGLT-2i, sodium-glucose cotransporter 2 inhibitor; TnT, troponin T.

Metric data are given as mean ± standard deviation or median (interquartile range) and categorical data as counts (percentage).

*p*-values derived from the Wilcoxon signed–rank test with continuity correction for metric variables and from the McNemar test for categorical and binary variables.

a Data missing in 1 patient.

b Data missing in 2 patients.

c Missing data in 4 patients.

d Missing data in 5 patients.

e Data missing in 7 patients.

f Data missing in 13 patients.Statistically significant *p*-values <0.05 marked in bold.

**Figure 2:**
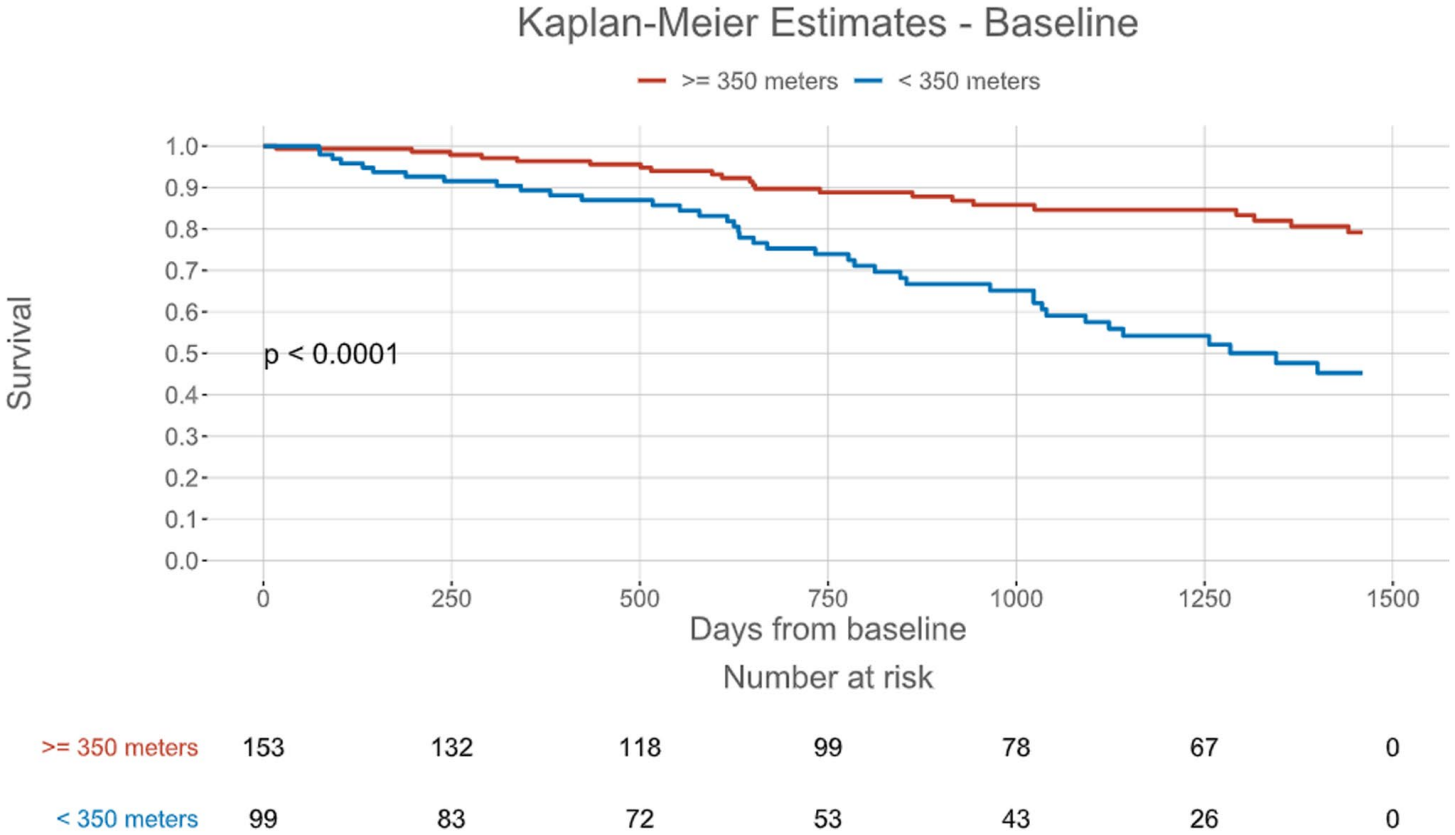
Kaplan-Meier estimates for survival stratified by the six-minute walk test distance threshold of < 350 meters from baseline.

**Figure 3:**
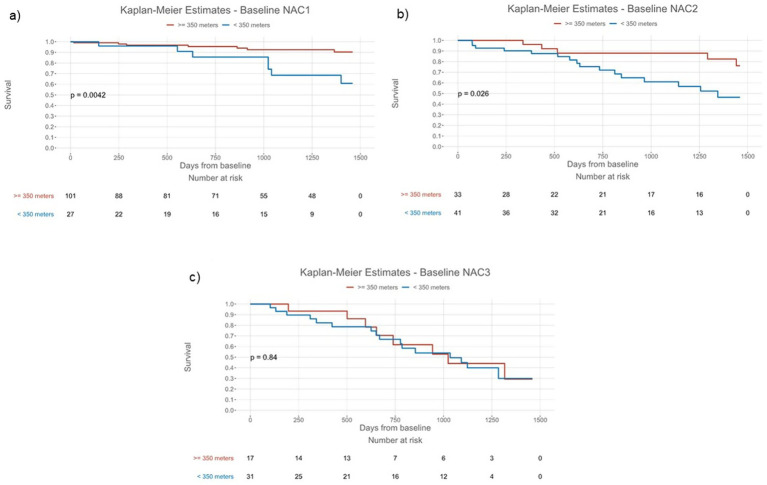
Kaplan-Meier estimates for survival stratified by the six-minute walk test distance threshold of < 350 meters in National Amyloidosis Centre stages 1 **(a)**, 2 **(b)**, and 3 **(c)** sub-cohorts from baseline; *NAC indicates National Amyloidosis Centre stage*.

The 6MWD at baseline was significantly associated with all-cause mortality [hazard ratio (HR) per 50 ms: 0.744, 95% confidence interval (CI): 0.673–0.827, *p* < 0.001]. When applying a 6MWD cutoff of < 350 m at baseline, patients had an approximately 3-fold risk of mortality (HR: 3.285, 95% CI: 1.944–5.551, *p* < 0.001), as shown in Table 2 and Figure 2. When adjusted for NAC stage, a significant association with all-cause mortality was demonstrated for the 6MWD and its cutoff at < 350 m (HR: 2.298, 95% CI: 1.286–4.104, *p* = 0.005). The log-rank test demonstrated significant deviation of the compared cohorts for patients in NAC stage 1 (*p* = 0.004) and 2 (*p* = 0.026), but not those in NAC stage 3 (Figure 3). In the linear regression analysis of baseline characteristics, age, troponin T, and eGFR were significantly associated with the *Δ* 6MWD.

**Table 2:** Cox regression for all-cause mortality from baseline.

Variable	Hazard ratio	95% confidence interval	*p*-value	Hazard ratio	95% confidence interval	*p*-value
	Crude			NAC adjusted		
6MWD, per 1 m	0.994	0.992–0.996	**<0.001**	0.996	0.994–0.998	**<0.001**
6MWD, per 50 m	0.744	0.673–0.827	**<0.001**	0.814	0.725–0.914	**<0.001**
6MWD, < 350 m	3.285	1.944–5.551	**<0.001**	2.298	1.286–4.104	**0.005**

Model statistics	
Number of events, *n* (%)	61 (24.2%)

6MWD indicates 6-min walk test distance; NAC, United Kingdom National Amyloidosis Centre stage. Statistically significant *p*-values <0.05 marked in bold.

### 3.2 Follow-up

A total of 190 patients completed a 1-year follow-up. Statistically significant changes between baseline and follow-up were observed for body mass index, levels of troponin T, and markers of kidney function. In addition, changes in patients’ background medication were observed (Table 1). Out of the 62 patients who did not complete follow-up, one (1.6%) patient died before completing follow-up, while all other patients (*n* = 61, 98.4%) were lost to follow-up.

Table 3 shows the Cox regression analysis from follow-up, where the 6MWD also demonstrated a significant association with the primary endpoint. A 6MWD of less than 350 m implied a 5.517-fold (95% CI 2.610–11.664, *p* < 0.001) risk of death from any cause.

**Table 3:** Cox regression analysis for all-cause mortality from follow-up.

Variable	Hazard ratio	95% Confidence interval	*p-*value	Hazard ratio	95% Confidence interval	*p*-value
	Crude			NAC adjusted		
6MWD, per +1 m	0.993	0.990–0.995	**<0.001**	0.995	0.992–0.998	**<0.001**
6MWD, per +50 m	0.697	0.614–0.794	**<0.001**	0.778	0.676–0.896	**<0.001**
6MWD, < 350 m	5.517	2.610–11.664	**<0.001**	2.912	1.279–6.629	**0.011**
Δ 6MWD, per +1 m	0.995	0.992–0.999	**0.011**	0.996	0.992–1.001	0.083
Δ 6MWD, < −35 m	1.831	0.968–3.462	0.063	1.573	0.811–3.050	0.180
Δ 6MWD, per +1%	0.984	0.971–0.996	**0.012**	0.992	0.979–1.005	0.237
Δ 6MWD, < −5%	1.881	0.988–3.582	0.055	1.326	0.657–2.676	0.431

Model statistics	
Number of events, *n* (%)	47 (24.7%)

Δindicates change in; 6MWD, 6-min walk test distance; NAC, United Kingdom National Amyloidosis. Statistically significant *p*-values <0.05 marked in bold.

When patients were stratified by tertiles of Δ 6MWD between baseline and follow-up (Δ ≤ −40, **≤ +** 15, > + 15), we found differences in NAC stage and biomarkers of heart failure. Follow-up characteristics are shown in Table 4. While the Δ 6MWD was also associated with mortality, the thresholds of −35 m and −5%, respectively, failed to reach the pre-defined level of statistical significance.

**Table 4:** Patient cohorts’ follow-up characteristics stratified by tertiles of change in the 6-min walk test distance.

Variable	Δ 6MWD tertile 1	Δ 6MWD tertile 2	Δ 6MWD tertile 3	*p*-value
	(*n* = 59)	(*n* = 55)	(*n* = 56)	
Δ 6MWD, m, border	≤ − 40	≤ + 15	> + 15	
Time to follow-up, months, median (IQR)	13 (12–15)	12 (8–16)	13 (10–14)	0.534
6MWD, m, mean (SD)	288 (117)	381 (124)	424 (134)	**<0.001**
Δ 6MWD, m, mean (SD)	−101 (61)	−10 (14)	73 (50)	**<0.001**
Δ 6MWD absolute threshold, *n* (%)	57 (100%)	2 (3.6%)	0 (0.0%)	**<0.001**
Δ 6MWD, %, mean (SD)	−27.8% (16.3%)	−2.5% (4.1%)	24.7% (20.3%)	**<0.001**
Δ 6MWD relative threshold, *n* (%)	57 (100%)	17 (30.9%)	0 (0.0%)	**<0.001**
Primary endpoint, *n* (%)	18 (31.6%)	16 (29.1%)	4 (7.1%)	**0.003**
Time to primary endpoint, months, median (IQR)	14.1 (8.9–16.5)	13.0 (9.7–19.5)	8.9 (5.5–11.3)	0.278
NT-proBNP, pg./mL, median (IQR)	2,593 (1633–5,302)	2,546 (1661–4,647)	1,533 (823–2,698)	**<0.001**
Δ NT-proBNP, pg(mL), median (IQR)	67 (−648–1,247)	271 (−250–1,262)	−18 (−1,058–295)	**0.029**
TnT, ng/L, median (IQR)	59.5 (38.0–89.0)	54.5 (33.5–75.0)	43.0 (28.0–54.0)	**0.006**
Δ TnT, ng/L, median (IQR)	3.0 (−2.0–22.5)	1.0 (−3.0–8.0)	−1.0 (−6.0–1.5)	**0.011**
eGFR, mL/min/1.73 m^2^, mean ± SD	48.1 ± 19.5	51.1 ± 18.7	59.6 ± 17.7	**0.002**
Δ eGFR, mL/min/1.73 m^2^, mean ± SD	−4.4 ± 9.8	−3.9 ± 10.4	−3.9 ± 12.6	0.746
NAC, *n* (%)				**<0.001**
1	23 (41.1%)	21 (40.4%)	38 (67.9%)	
2	17 (30.4%)	20 (38.5%)	15 (26.8%)	
3	16 (28.6%)	11 (21.2%)	3 (5.4%)	
Tafamidis, *n* (%)	57 (100%)	35 (64.8%)	50 (89.2%)	**<0.001**
Started on tafamidis, *n* (%)	53 (93.0%)	35 (64.8%)	47 (83.9%)	**<0.001**
Time on tafamidis, months, median (IQR)	11.4 (6.6–13.1)	6.6 (6.0–11.6)	11.3 (6.4–12.9)	**0.034**
Loop diuretics, *n* (%)	43 (76.8%)	34 (65.4%)	31 (56.4%)	0.074
Loop diuretic dose, mg/kg, median (IQR)	0.345 (0.000–0.818)	0.278 (0.000–0.893)	0.167 (0.000–0.382)	**0.022**
Loop diuretic escalation, *n* (%)	21 (42.0%)	17 (36.2%)	10 (19.2%)	**0.045**

6MWD indicates 6-min walk test distance; eGFR, estimated glomerular filtration rate; IQR, interquartile range; NAC, United Kingdom National Amyloidosis Centre staging system; NT-proBNP, N-terminal prohormone of B-type natriuretic peptide; SD, standard deviation; TnT, troponin T.

Metric data are given as mean ± standard deviation or median (interquartile range), and categorical data as counts (percentage).

*p*-values derived from the Kruskal–Wallis test for metric and the chi-squared test for binary/categorical variables. Statistically significant *p*-values <0.05 marked in bold.

**Figure 4:**
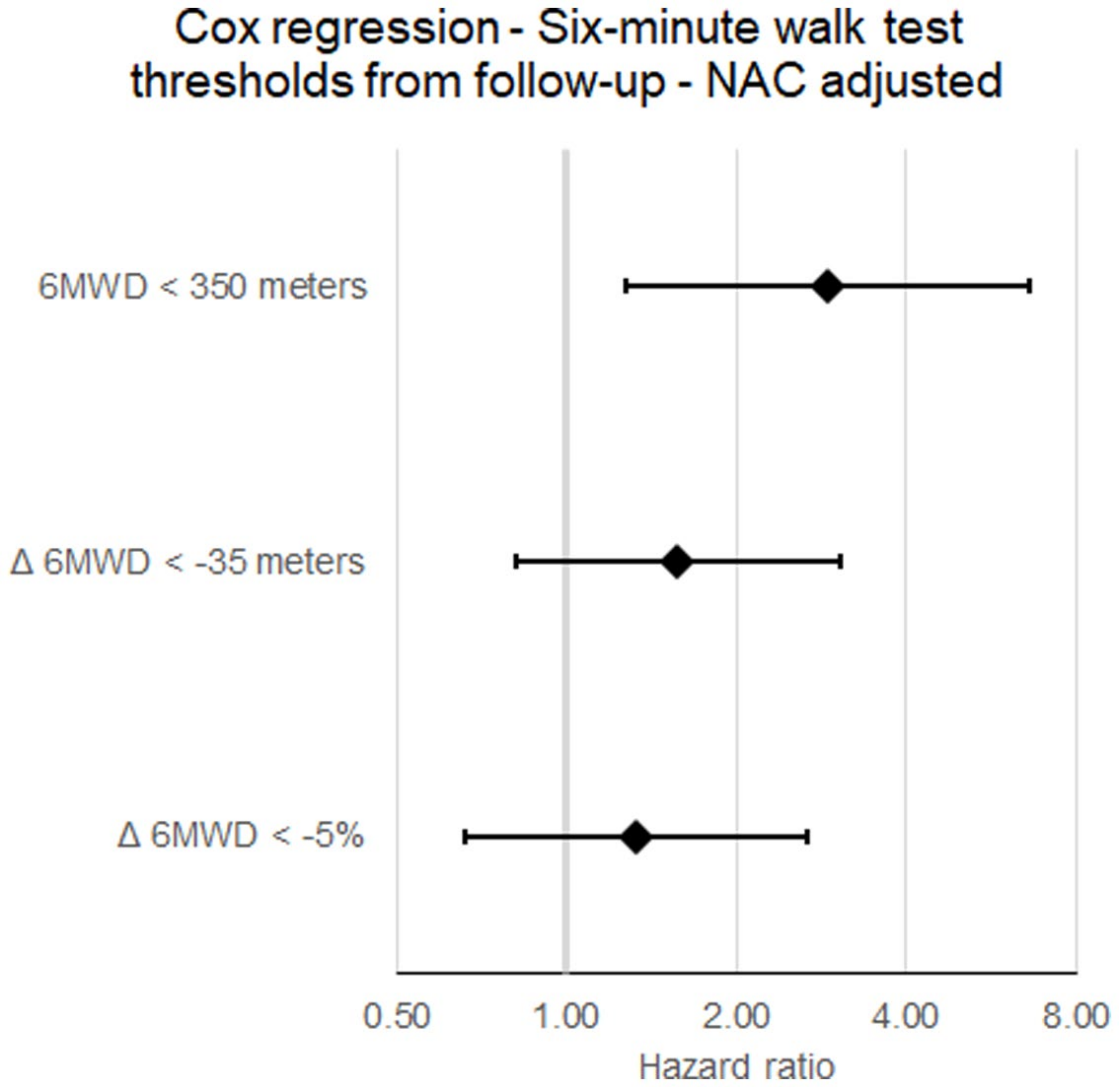
Forest plot of six-minute walk test distance thresholds at follow-up adjusted for National Amyloidosis Centre stage; Δ *indicates change in; 6MWD, six-minute walk test distance; NAC, National Amyloidosis Centre stage*.

After adjusting for NAC stage, neither the crude Δ 6MWD nor the thresholds remained significantly associated with mortality, while the 6MWD at follow-up was still strongly significantly associated with death from any cause (HR for 6MWD < 350 m: 2.912, 95% CI: 1.279–6.629, *p* = 0.011, Figure 4). In an additional Cox regression model adjusting the *Δ* 6MWD thresholds for changes in amyloid-specific treatment status, both the absolute threshold of −35 m (HR: 2.726, 95% CI: 1.287–5.777, *p* = 0.009) and −5% (HR: 3.577, 95% CI: 1.527–8.382, *p* = 0.003) remained significantly associated with outcomes.

Among follow-up characteristics, Δ troponin T—but neither Δ N-terminal prohormone of B-type natriuretic peptide (NT-proBNP) nor Δ eGFR—was a significant determinant of the Δ 6MWD. In a multivariate model, no baseline characteristics remained significantly associated with Δ 6MWD. Regression analyses are shown in Supplementary Table S1.

## 4 Discussion

The results of our analysis support the applicability of the 6MWT in patients with ATTR-CM receiving disease-modifying therapy for estimating prognosis throughout the disease course and externally validate the previously proposed 6MWD threshold of < 350 m (8). The association between 6MWD and all-cause mortality persisted after adjusting for the ATTR-CM-specific NAC staging system. However, our results do not support the use of changes in 6MWD in patients recently started on disease-modifying therapy for the estimation of prognosis.

Notably, this study is dedicated to assessing the prognostic implications of the 6MWT in patients with ATTR-CM who receive amyloid-specific disease-modifying treatment. Therefore, most of our patients were started on disease-modifying therapy during the study period, while due to United Kingdom regulation, tafamidis was not yet widely available to patients studied by Ioannou et al. (8) have censored patients with the prescription of amyloid-specific treatments, such as transthyretin stabilizers or silencers, in their main analysis, thus limiting the applicability of their findings to current real-world ATTR-CM patients.

Therefore, in our large single-center prospective ATTR-CM cohort, which outnumbered placebo cohorts of the phase III trials of both tafamidis (11) and acoramidis (16) (*n* = 252, ATTR-ACT: *n* = 177, ATTRibute-CM: *n* = 211), we demonstrated that, even after adjusting for NAC stage, which incorporates NT-proBNP and eGFR, the 6MWD was independently associated with all-cause mortality.

In this real-world cohort, the threshold of < 350 m was associated with approximately triple the risk of mortality, even exceeding the relative risk of 2.2-fold calculated by Ioannou et al. (8) After adjusting in a multivariate model, a distance below 350 m still implied a ~2.4-fold increase in risk in our cohort. In patients receiving contemporary standard of care, the association of the 6MWT with mortality was even more pronounced; as a result, below the threshold of 350 m implied a HR of approximately 5.5 in the univariate analysis. However, as compared to the population observed by Ioannou et al., the proportion of variant transthyretin amyloid (ATTRv) patients was smaller in our cohort (<8% vs. >25%). As this most likely reflects different regional distributions of ATTRv gene carriers, it should also be taken into consideration when interpreting the present study’s results.

In our cohort, recruited between March 2012 and July 2024, the majority of patients were diagnosed and included after the European Medicines Agency approved tafamidis in 2020 and were started on disease-modifying therapy as soon as possible. Consequently, most of our patients were started on disease-modifying therapy during the study period, however, tafamidis was not yet available due to regulation restrictions in the United Kingdom.

We failed to demonstrate a significant association between the cutoffs for Δ 6MWD between baseline and follow-up in our patients, even in the univariate analysis. Only in an experimental Cox regression analysis adjusting for changes in disease-specific treatment status did these thresholds show a significant association with all-cause mortality. This finding supports the notion that a change in treatment status is a crucial confounder for analyzing the results of repeated 6MWTs. Concurrently, evaluation of the Δ 6MWD may be insufficient to adequately estimate prognosis in However, the Δ 6MWD may be a suitable marker of disease progression or treatment response after a certain run-in phase of amyloid-specific disease-modifying therapy and under unchanged supporting therapy. Beyond confounding by changes in treatment status, the 6MWD is also criticized for being influenced by orthopedic and neurologic limitations, including but not limited to arthrosis and polyneuropathy (17). While both of these entities may be seen at least partially due to transthyretin amyloidosis, they may be considered endemic in an elderly population. A potentially more objective assessment of maximal physical capacity would be cardio-pulmonary exercise testing and evaluation of maximum oxygen consumption (17, 18).

Interestingly, in the linear regression analysis, the *Δ* 6MWD seems to be driven mostly by age, troponin T, and eGFR, but not NT-proBNP. In the multivariate analysis, no baseline parameter remained a significant determinant of the Δ 6MWD. Even among follow-up parameters, the Δ troponin T was the only parameter associated with the Δ 6MWD. It may be speculated that NT-proBNP could be too sensitive to short-term changes in volume status, while troponin T could be interpreted as a more reliable indicator of long-term myocardial damage.

Currently, the most widely used disease staging system in ATTR-CM relies exclusively on laboratory assessments, most prominently NT-proBNP and eGFR (6, 19). The incorporation of measurements beyond biomarkers may aid a more holistic approach to patient risk stratification for various aspects of disease. As the 6MWT and patient-reported outcome measurement tools have been demonstrated to be associated with outcomes independent of laboratory assessments (3, 8), the incorporation of such easily applicable tools into routine practice may be warranted. Interestingly, in our sub-cohort of patients in NAC stage 3, the association of 6MWD of < 350 m was practically non-existent, while for NAC stages 1 and 2, survival was markedly better in patients exceeding 350 m in 6MWD. While this contradicts previous subgroup analyses (8), it suggests that, in patients with very advanced disease, consideration of alternative measurements may be warranted.

From the pre-existing randomized controlled trials (11, 16, 20) and the established therapeutics’ pathophysiologic effects, it may be assumed that disease-specific treatment slows disease progression but is currently unable to improve physical capabilities. Therefore, our results may be surprising, as a relevant proportion of patients improve in the 6MWT. However, this finding is in line with a previous study in our cohort where treatment with tafamidis resulted in slight increases in physical performance in the short-term (4).

Importantly, both disease-specific questionnaires and evaluations of physical performance, like the 6MWT, can be performed without complex and expensive laboratory equipment and are easily available even in non-academic or budget-constrained settings. In comparison to risk stratification relying on NT-proBNP and eGFR alone, the 6MWT as an assessment of everyday physical ability may be superior in assessing the disease’s impact on patients’ quality of life, which is significantly associated with physical capabilities. In contrast to routine laboratory assessments, the performance of the 6MWT is also dependent on non-cardiac factors.

### 4.1 Future directions

As the cumulative evidence available suggests a valuable role for the 6MWT in risk stratification, future studies and large-scale registry analyses may aid in the development of a refined staging system for ATTR-CM. Recently, the most widely used NAC staging system has been expanded with an additional stage at the top end for the most severely affected (19), the addition of physical performance measures may further refine risk stratification, supposedly especially in earlier disease stages, such as in pulmonary arterial hypertension (21). While the relative simplicity of the 6MWT makes this test a promising candidate, alternative measurements of physical performance, including cardiopulmonary exercise testing (4) and the 1-min sit-to-stand test (22), may also be considered, especially in the most advanced diseases, as we have shown. While there is some literature supporting cardiopulmonary exercise testing for risk stratification in ATTR-CM (4), the 1-min sit-to-stand test (22) remains to be evaluated in patients with ATTR-CM. Finally, these results may have important implications for clinical risk evaluation and the design of future trials, potentially supporting earlier decisions to change disease-specific therapeutics as soon as additional agents for the treatment of ATTR-CM become available.

### 4.2 Limitations

Due to the retrospective nature of our analysis, certain inherent limitations need to be considered. First, biases unbeknown to the investigators may not be fully excluded, especially with regard to the prescription of disease-specific treatment, which has also changed over time as the availability of therapeutic agents has increased.

Second, as the prospective registry was conducted at a single center, we could not validate our findings, which should ideally be verified in a multi-center, multi-national cohort of patients with ATTR-CM receiving disease-modifying therapeutics (8). Naturally, comparisons between our cohort and the significantly larger NAC cohort (8) need to be interpreted with the utmost caution due to differences in population characteristics, treatment status, follow-up time, and referral patterns, as we have discussed.

Furthermore, patients who are unable to perform the 6MWT due to physical impairment, recent surgery, or injury represent a significant proportion of patients. However, the exact reasons for not performing a 6MWT were not always available in our cohort. This may limit the accurate characterization of these patients, and it may be assumed that other methods of assessing submaximal physical performance are required in this subgroup of patients.

Finally, a significant proportion of patients did not complete follow-up. Although only one patient died during this period, this still represents a relevant proportion of patients and may have introduced bias in our analysis of Δ 6MWD.

## 5 Conclusion

Performance in the 6MWT is independently associated with all-cause mortality in patients with ATTR-CM, irrespective of treatment status. A baseline 6MWD of < 350 m is associated with a ~3-fold increased risk of death from any cause. However, our results do not support the use of the Δ 6MWD as an indicator for adverse outcomes in patients whose disease-modifying therapy was recently adapted.

## Data Availability

The data supporting this manuscript may be shared upon reasonable request to the corresponding author.
