# Supplementary material for: The 6-min walk test in transthyretin cardiac amyloidosis: prognostic utility put to the real-world test
Source: Front Med (Lausanne). 2025 Sep 8;12:1639586. doi: 10.3389/fmed.2025.1639586 (PMC12450996; doi:10.3389/fmed.2025.1639586)
Supplement: Supplementary file 1 [file Table_1.docx]

**APPENDIX**

**Supplementary table S1. Univariate and multivariate linear regression model for the delta six-minute walk test distance.**

| **Variable** | **Regression coefficient** | **95% Confidence interval** | ***P-value*** | **Regression coefficient** | **95% Confidence interval** | ***P-value*** |
| --- | --- | --- | --- | --- | --- | --- |
|  | **Univariate analysis** | | | **Multivariate analysis** | | |
| **Baseline** |  | | |  | | |
| Age | -1.906 | -3.627 – -0.185 | ***0.030*** | -1.45 | -3.47 – 0.575 | *0.159* |
| Female | 5.223 | -30.9 – 41.3 | *0.776* |  |  |  |
| ATTRv | 14.9 | -35.7 – 65.6 | *0.562* |  |  |  |
| BMI | 0.607 | -0.690 – 1.902 | *0.357* |  |  |  |
| Heart rate | 0.775 | -0.191 – 1.740 | *0.115* |  |  |  |
| BP systolic | -0.025 | -0.737 – 0.686 | *0.944* |  |  |  |
| BP diastolic | 0.108 | -0.157 – 0.373 | *0.421* |  |  |  |
| NT-proBNP | -0.001 | -0.004 – 0.002 | *0.423* |  |  |  |
| Troponin T | -0.618 | -1.075 – -0.161 | ***0.008*** | -0.487 | -1.01 – 0.038 | *0.069* |
| eGFR | 0.706 | 0.043 – 1.369 | ***0.037*** | 0.068 | -0.763 – 0.898 | *0.873* |
| C-reactive protein | -1.914 | -9.563 – 5.735 | *0.612* |  |  |  |
| AF | 4.35 | -22.2 – 30.9 | *0.747* |  |  |  |
| sCAD | 3.00 | -28.2 – 34.2 | *0.850* |  |  |  |
| aHTN | -22.6 | -51.5 – 6.3 | *0.125* |  |  |  |
| Diabetes mellitus | -20.5 | -51.2 – 10.3 | *0.191* |  |  |  |
| **Follow-up** |  |  |  |  |  |  |
| Δ NT-proBNP | -0.003 | -0.007 – 0.001 | *0.092* |  |  |  |
| Δ Troponin t | -2.06 | -3.21 – -0.905 | ***<0.001*** | -2.06 | -3.21 – -0.905 | ***<0.001*** |
| Δ eGFR | 0.718 | -0.504 – 1.940 | *0.248* |  |  |  |

*AF indicates atrial fibrillation; aHTN, arterial hypertension; ATTRv, variant transthyretin amyloid; BMI, body-mass index; BP, blood pressure; eGFR, estimated glomerular filtration rate; NT-proBNP, N-terminal prohormone of B-type natriuretic peptide; sCAD, significant coronary artery disease;*
